# Secreted Frizzled-related protein 4 (sFRP4) chemo-sensitizes cancer stem cells derived from human breast, prostate, and ovary tumor cell lines

**DOI:** 10.1038/s41598-017-02256-4

**Published:** 2017-05-23

**Authors:** A. Deshmukh, S. Kumar, F. Arfuso, P. Newsholme, A. Dharmarajan

**Affiliations:** 1 Stem Cell and Cancer Biology Laboratory, School of Biomedical Sciences, Curtin Health Innovation Research Institute, Curtin University, Perth, WA Australia; 2 School of Biomedical Sciences, Curtin Health Innovation Research Institute, Curtin University, Perth, WA Australia

## Abstract

This study investigated molecular signals essential to sustain cancer stem cells (CSCs) and assessed their activity in the presence of secreted frizzled-related protein 4 (sFRP4) alone or in combination with chemotherapeutic drugs. SFRP4 is a known Wnt antagonist, and is also pro-apoptotic and anti-angiogenic. Additionally, sFRP4 has been demonstrated to confer chemo-sensitization and improve chemotherapeutic efficacy. CSCs were isolated from breast, prostate, and ovary tumor cell lines, and characterized using tumor-specific markers such as CD44^+^/CD24^−^/CD133^+^. The post-transcription data from CSCs that have undergone combinatorial treatment with sFRP4 and chemotherapeutic drugs suggest downregulation of stemness genes and upregulation of pro-apoptotic markers. The post-translational modification of CSCs demonstrated a chemo-sensitization effect of sFRP4 when used in combination with tumor-specific drugs. SFRP4 in combination with doxorubicin/cisplatin reduced the proliferative capacity of the CSC population *in vitro*. Wnt/β-catenin signaling is important for proliferation and self-renewal of CSCs in association with human tumorigenesis. The silencing of this signaling pathway by the application of sFRP4 suggests potential for improved *in vivo* chemo-responses.

## Introduction

Chemotherapy, along with radiotherapy and hormone therapy, is the most common treatment for cancer. Due to the side effects of treatment and chemo-resistance of tumor cells, researchers have shifted their focus to more site-specific treatments in order to achieve better patient outcomes^1^. Over the past decade, a critical role of a small subset of tumor cells, known as cancer stem cells (CSCs), was established in tumor relapse and propagation^2, 3^. Most solid tumors, including breast, brain, prostate, ovary, mesothelioma, and colon cancer contain this small subset of self-renewing, tumor initiating cells^4^. Conventional anti-cancer therapies inhibit/kill the bulk of the heterogeneous tumor mass, resulting in tumor shrinkage. However, it has been suggested that later, the CSCs differentiate into tumor cells and are responsible for tumor relapse^5, 6^. CSCs are characterized by their tumor forming ability and expression of high levels of ATP-binding cassette drug transporters (ABCG2), cell adhesion molecules (CD44), and anchorage independent cell survival proteins (Cyclin D1), which are collectively responsible for chemo-resistance^7–9^. In human breast, ovary, and prostate cancers, several CSC populations have been identified using cell surface markers (CD44^+^/CD133^+^/CD24^−/low^); these CSCs have shown a high clonal, invasive, and metastatic capacity, leading to resistance to radio-therapy, chemotherapeutic drugs (doxorubicin and cisplatin), and other target-specific therapy^10–12^.

CSCs possess high capacity for tumor propagation and metastasis^13–15^, which causes more than 90% of cancer-related deaths. The molecular mechanism of CSCs regulating metastasis is not completely understood; however, the invasive metastatic cascade involves circulation of cancer cells through the surrounding extracellular matrix in a multistep cellular operation.

The development and maintenance of CSCs is controlled by several signaling pathways such as Wnt and Notch. The Wnt pathway is known to mediate the self-renewal capacity of CSCs through modulation of β-catenin/TCF transcription factors. There is evidence suggesting a Wnt signaling role in CSC maintenance (as seen in murine models and humans) of non-melanoma cutaneous tumor, where CSCs are maintained by Wnt/β-catenin signaling^16^. The interactions of Wnt proteins to the receptor complex can be inhibited by binding of the ligands to endogenous Wnt antagonists such as secreted frizzled-related proteins (sFRPs)^17^. SFRP4 is one of the prominent isoforms with the capacity to chemo-sensitize tumor cells to chemotherapeutics^18, 19^. Chemo-sensitization of CSCs by sFRP4 has the potential to decrease the required chemotherapeutic load to facilitate tumor resolution.

## Results

### Tumor derived CSCs characterization

Spheroids obtained for CSC isolation were characterized for the expression of tumor-specific CSC markers CD44^+^ / CD24^−/low^ for breast CSCs, and CD133^+^/CD44^+^ for prostate and ovarian CSCs (Table 1), by using flow cytometry. The combinatorial treatment showed significant reduction in the CSC marker population in all cell line-derived CSCs; although in A2780 prostate CSCs, cisplatin treatment showed phenotype switching to CD44^+^ positive cells and only reduced the CD133^+^ population; however, this switching did not affect the inhibitory effect of combinatorial treatment (see Supplementary Figure 1). The characterized CSCs were further used for functional analysis.

**Table 1:** Effect of sFRP4 on CSCs characterization.

Treatment	Surface markers	CD44^+^/CD24^−^ (%)		CD133^+^/CD44^+^ (%)				
		MDA231	MCF7	PC3	LnCap	A2780 P	A2780 ADR	A2780 CIS
*Untreated*		58.1	36.7	24.3	62.7	2.85	19.5	2.72
*sFRP4*		32.3	17.2	17.2	44.1	2.99	16.8	2.21
*Dox*./*Cisplatin*		28.5	14.9	22.1	47.5	9.29	16	1.8
*sFRP4* + *Dox./Cisplatin*		**25**	**1.68**	**9.16**	**10.4**	**1.88**	**15.1**	**1.23**

Using flow cytometry, Statistical analysis of CSC markers post treatment. Data are means ± percentage from 3 independent experiments.

### SFRP4 in combination with doxorubicin/cisplatin reduces the sphere forming capacity of CSCs

**Figure 1:**
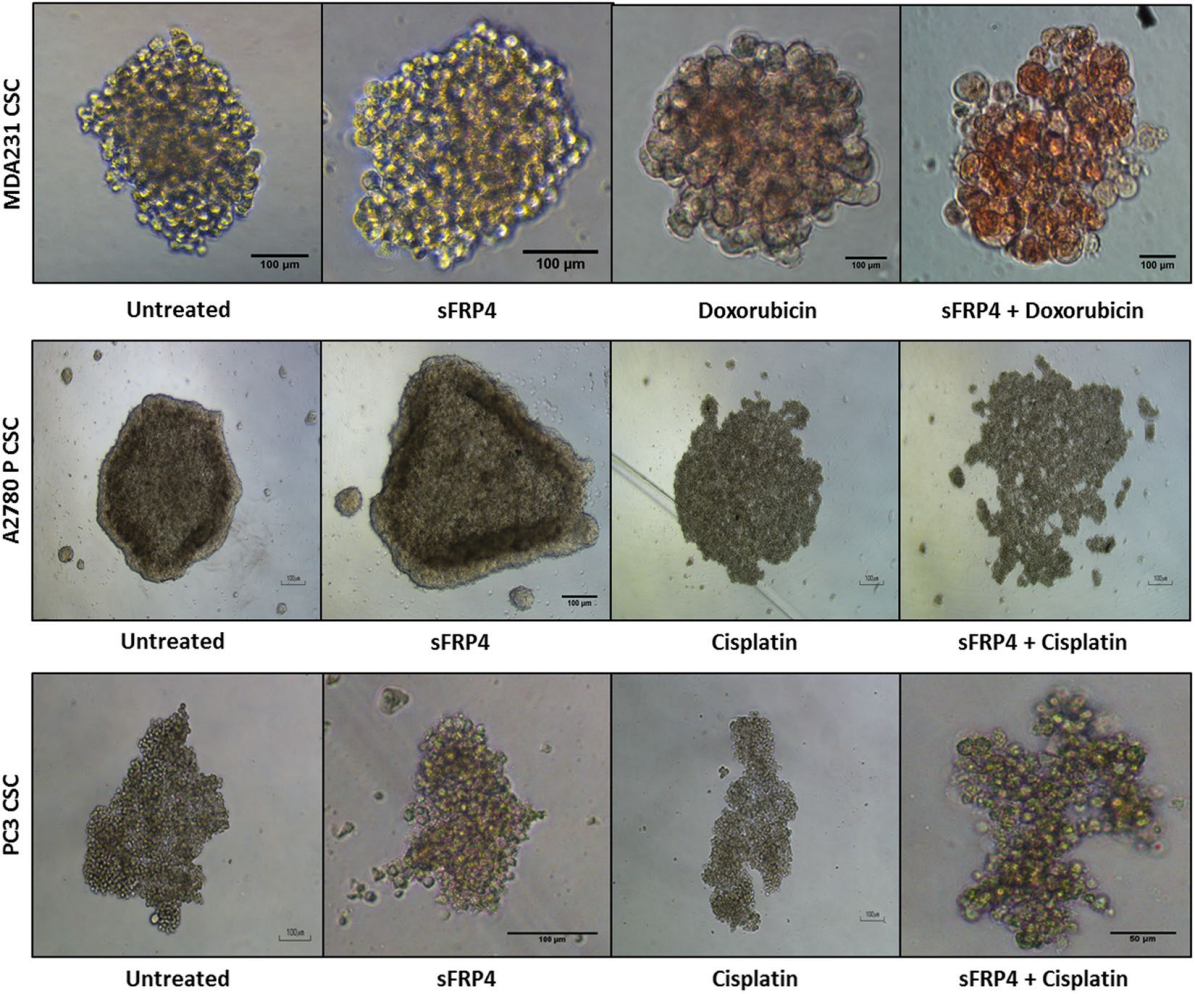
Effect of sFRP4 on CSC Morphology: CSCs were isolated from breast, ovary, and prostate tumor cell lines and treated with sFRP4 (250 pg) with chemotherapeutic drugs (doxorubicin 5 μM/cisplatin 30 μM). The combinatorial treatment shows the disruption of the CSC sphere. (Scale bar: 100 μm). Cells images are representative from all the experiments.

**Figure 2:**
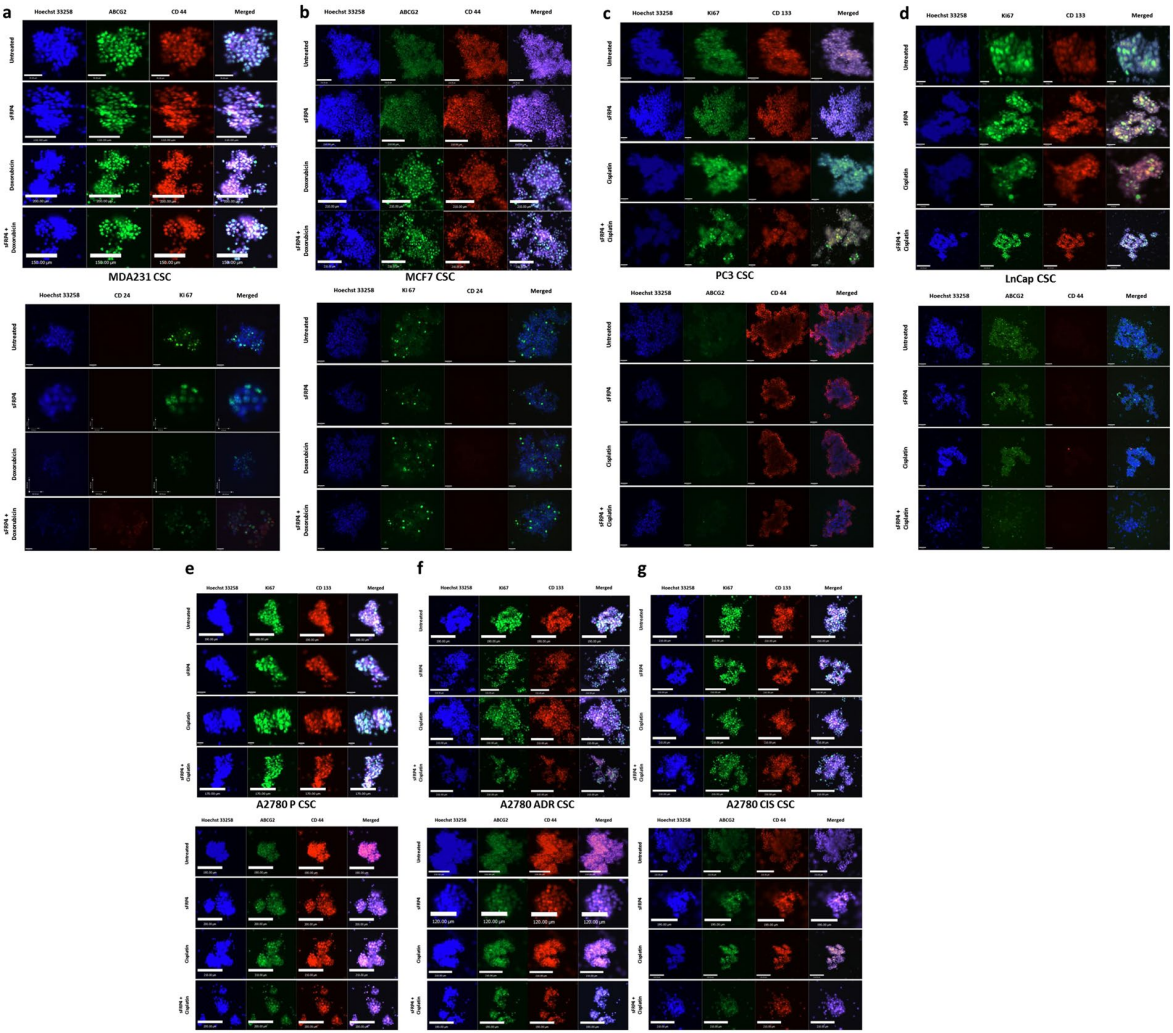
Effect of sFRP4 on CSC cell surface markers: Using Immunofluorescence, CD44^+^/CD24^−^/CD133^+^/Ki67/ABCG2 were detected in all the tumor cell line-derived CSCs. sFRP4 in combination with doxorubicin/cisplatin showed disruption of the spheres. Nuclei were counterstained with Hoechst 33342 (blue). (**a,b**) CD44^+^/CD24^−^ breast tumor cell line CSCs (MDA231/MCF7). (**c,d**) Prostate tumor cell line derived CSCs. PC3 expressed CD44^+^/CD133^+^/Ki67/ABCG2. LnCap was negative for CD44^+^. (**e–g**) CD44^+^/CD133^+^/Ki67/ABCG2 observed in ovary tumor cell line-derived CSCs (A2780 P/CIS/ADR). Immunofluorescence images are representative from 3 independent experiments. (Scale bar: 100 μm).

The CSCs derived from breast, prostate, and ovary tumor cell lines were treated with sFRP4 (250 pg) and doxorubicin (5 μM)/cisplatin (30 μM) alone or in combination. The untreated spheroids remained intact, whereas the combinatorial treatment of sFPR4 and chemotherapeutic drugs showed disruption of spheroids post-treatment (Fig. 1), indicating sFRP4’s capacity to segregate the tumor spheres and allow chemotherapeutic drugs to inhibit tumor proliferation. This was further confirmed by immunofluorescence, where spheroids were labelled with CD44^+^/CD24^−/low^/ABCG2/KI67 for CSCs derived from breast tumors (Fig. 2a and b), and CD133^+^/CD44^+^/ABCG2/Ki67 for prostate (Fig. 2c and d) and ovarian tumors (Fig. 2e, f, and g); except for the prostate LnCap CSCs, which were not CD44^+^ (Fig. 2d). The combinatorial treatment showed sphere disruption and a reduction in surface receptor expression compared to sFRP4 and drugs alone, indicating the effect of sFRP4 in inhibiting the spheroids’ proliferative capacity.

### SFRP4 in combination with doxorubicin/cisplatin reduces CSC viability

**Figure 3:**
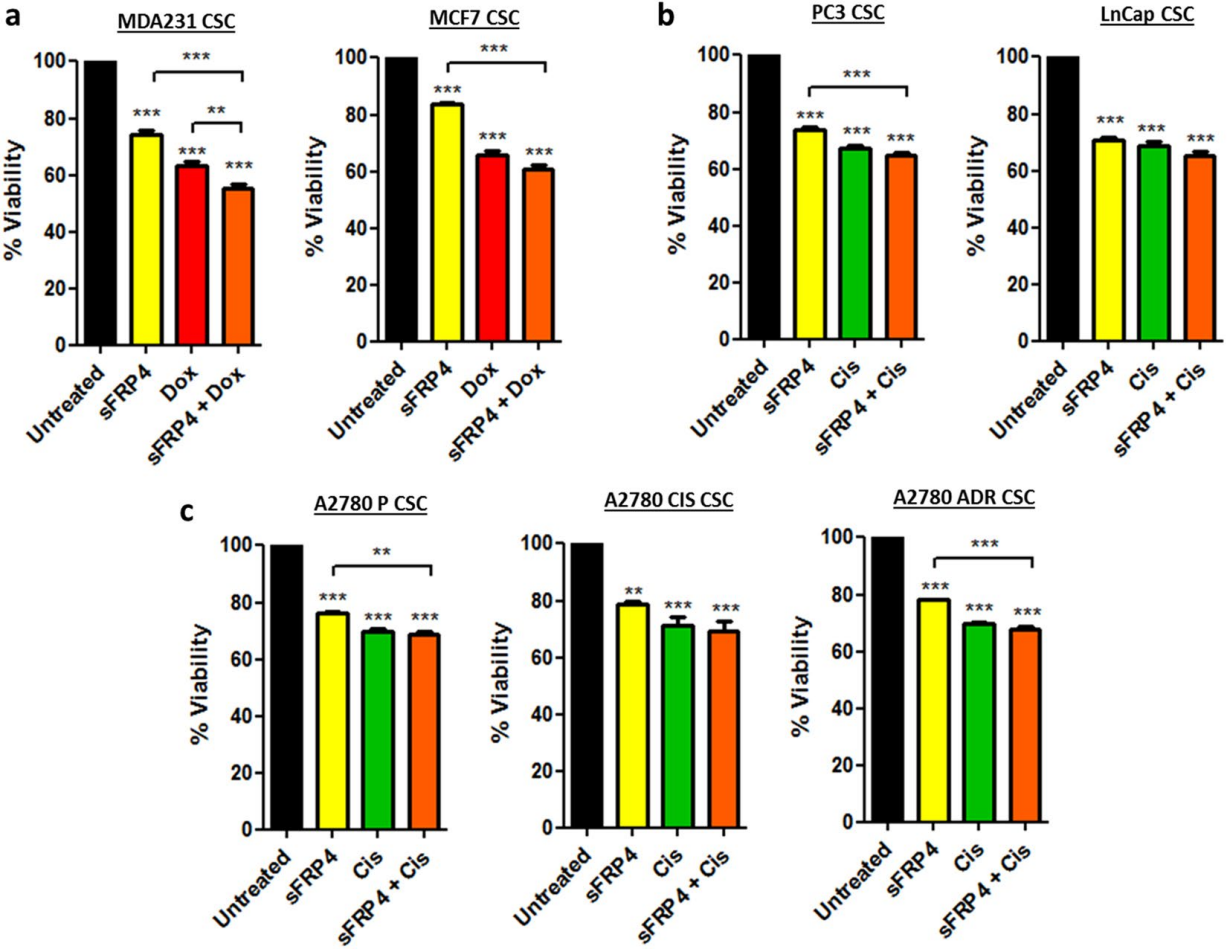
Effect of sFRP4 on CSC viability: Viability assay was performed by MTT after treatment of CSCs derived from (**a**) Breast tumor cell line-derived CSCs (MDA231/MCF7). (**b**) Prostate tumor cell line-derived CSCs (PC3/LnCap). (**c**) Ovary tumor cell line-derived CSCs (A2780 P/CIS/ADR) with sFRP4 alone or in combination with doxorubicin/cisplatin for 24 hr. Statistical analysis was performed using ANOVA for analysis variance with Bonferroni test for comparison showing significance as ***P < 0.001; **P < 0.01; *P < 0.05. Data are mean ± standard error of mean from 3 independent experiments.

Using an MTT assay, it was observed that the combinatorial treatment of sFRP4 and doxorubicin/cisplatin significantly inhibits the viability of CSCs (P < 0.001, n = 3) compared to sFRP4 or drugs alone. Similar patterns were observed in all the cell lines (Fig. 3). Therefore, this treatment combination was used for subsequent studies.

### SFRP4 and doxorubicin/cisplatin treatment downregulates the expression of CSC stemness genes

**Figure 4:**
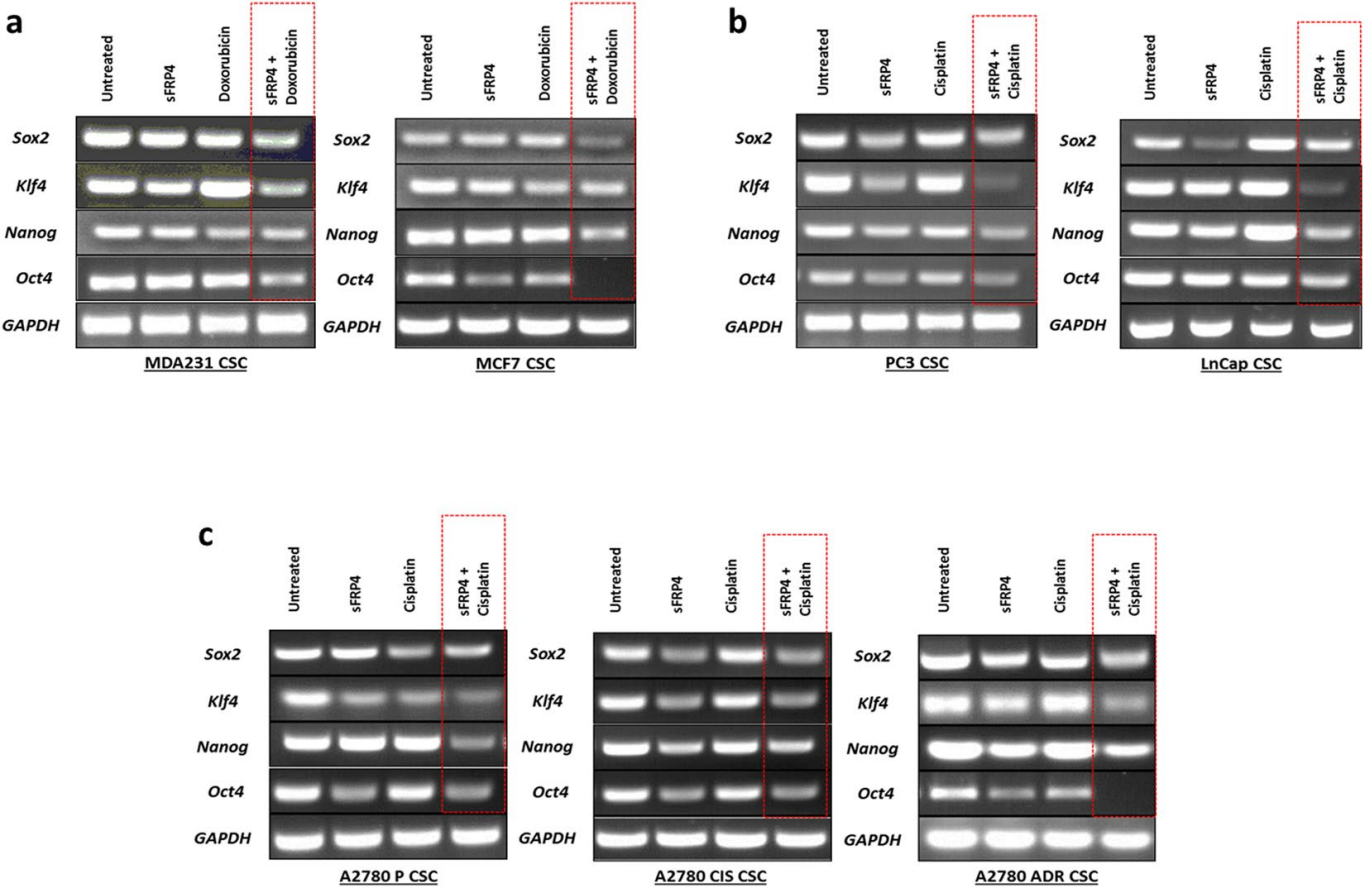
Effect of sFRP4 on CSC stemness gene expression: sFRP4 in combination with chemotherapeutic drugs (Dox/Cis.) reduced the expression of stemness-related genes, indicating loss of stem like expression and differentiation capacity. Semi-quantitative PCR images are representative of 3 experiments.

The stemness related genes *SOX2*, *Klf4*, *Nanog*, and *Oct4* are expressed in CSCs and are associated with tumor progression. Semi-quantitative PCR analysis showed the untreated CSCs expressing all the genes, but the treatment with sFRP4 alone or in combination with doxorubicin/cisplatin downregulated the expression of *SOX2*, *Klf4*, *Nanog*, and *Oct4* in all the cell line-derived CSCs. The combinatorial treatment showed maximum reduction of gene expression, indicating that sFRP4 in combination with chemotherapeutic drugs has the capacity to reverse the stem cell-like properties of CSCs (Fig. 4).

### SFRP4 mediates early apoptotic events in CSCs

**Figure 5:**
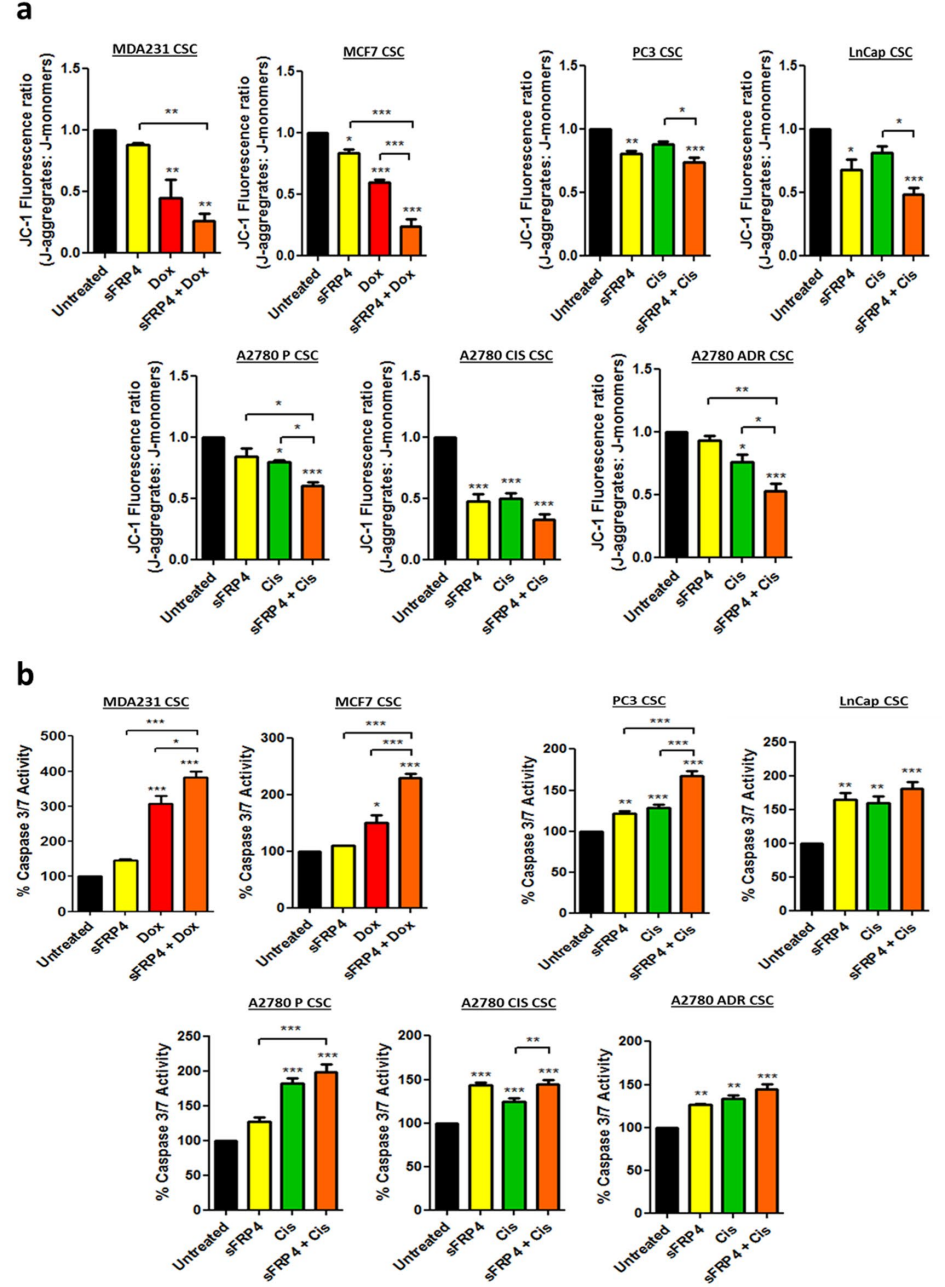
sFRP4 initiates early apoptotic events in CSCs. (**a**) Detection of JC-1: The JC-1 assay demonstrated a high mitochondrial depolarization in sFRP4, doxorubicin/cisplatin, and combination-treated CSCs, indicating early stage cell death and apoptotic response to the various drug treatments. The combinatorial treatment on CSCs showed maximum depolarization. (**b**) Detection of caspase-3 activity: Increasing amount of caspase-3 substrate indicates initiation of apoptosis. The caspase-3 activity (an indicator of late apoptosis) of CSCs was significantly upregulated in combinatorial treatment compared to untreated cells. Statistical analysis was performed using ANOVA for analysis variance with Bonferroni test for comparison showing significance as ***P < 0.001; **P < 0.01; *P < 0.05. Data are mean ± standard error of mean from 4 independent experiments.

The disruption of mitochondrial membrane potential was investigated by using JC-1 dye. Results from the JC-1 assay demonstrated a significant increase (p < 0.01) in mitochondrial depolarization after treatment with sFRP4, doxorubicin/cisplatin alone, and in combinatorial treatments compared to untreated control. In all cell line-derived CSCs, maximum depolarization was observed in combinatorial treatments, indicating early stage death and apoptotic response through sFRP4 (Fig. 5a). To further confirm the apoptotic role of sFRP4 in CSCs, we studied caspase 3 activity in CSCs derived from all cell lines, which indicated increased caspase 3 activity (p < 0.001) in the sFRP4 alone and combinatorial treatments in comparison to untreated cells (Fig. 5b).

### SFRP4 regulates protein expression in CSCs

**Figure 6:**
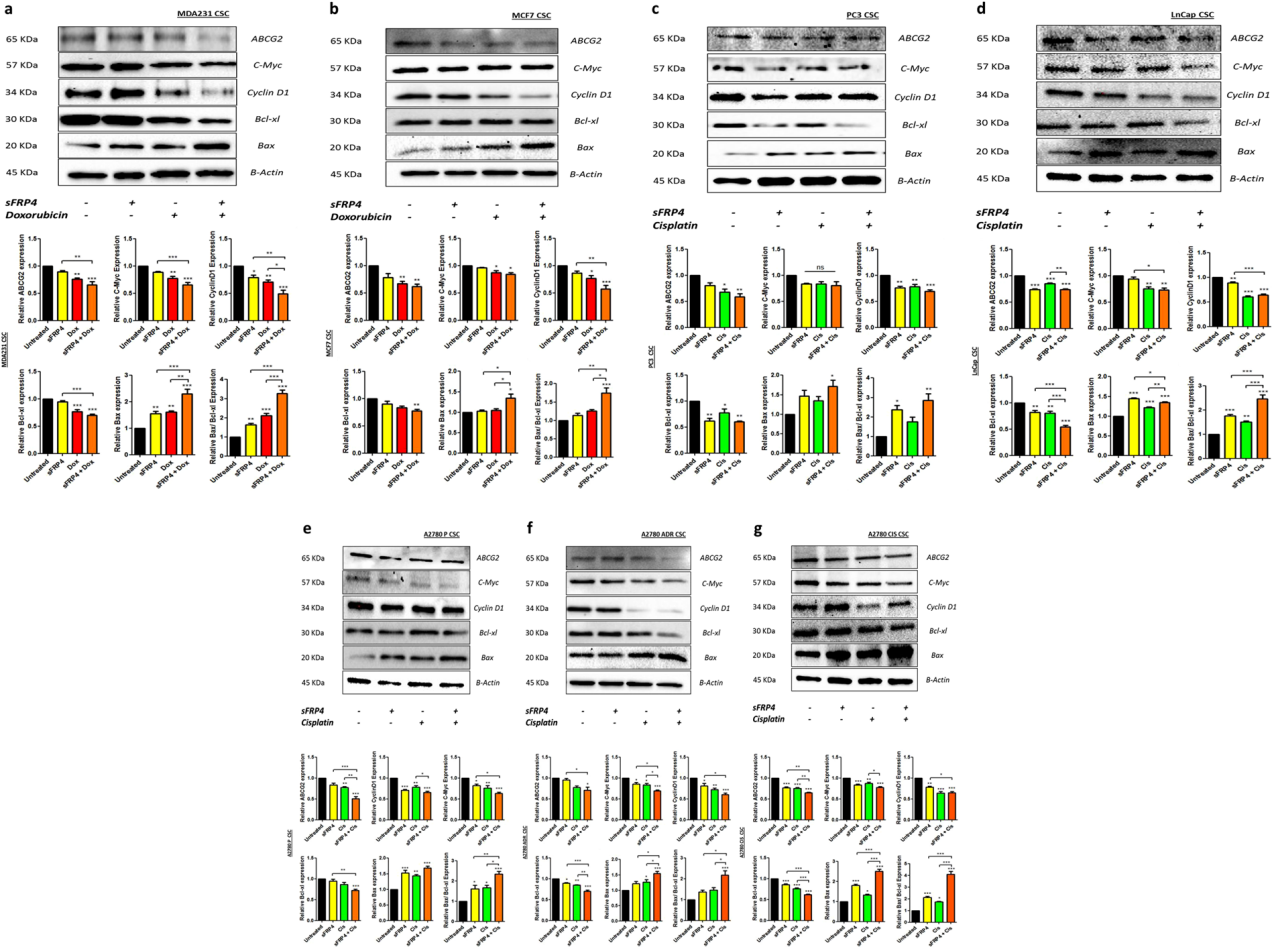
Effects of sFRP4 on protein expression levels: The combinatorial treatment of sFRP4 and chemotherapeutic drugs significantly upregulated the apoptotic protein (Bax) and downregulated cell survival (Cyclin D1) and oncogenes (*C-Myc*). (**a,b**) Breast tumor cell line-derived CSCs (MDA231/MCF7). (**c,d**) Prostate tumor cell line-derived CSCs (PC3/LnCap). (**e–g**) Ovary tumor cell line-derived CSCs (A2780 P/CIS/ADR). In combinatorial treatment, an elevated Bax/Bcl-xl ratio corresponded to elevated cell apoptosis. Statistical analysis was performed using ANOVA for analysis variance with Bonferroni test for comparison showing significance as ***P < 0.001; **P < 0.01; *P < 0.05. Blots and relative protein expressions are mean ± standard error of mean from 3 independent experiments.

Following sFRP4 treatment, we investigated the post-translational modifications in CSCs for ABC transporters (ABCG2), oncogenes (*c-Myc*), anchorage independent cell survival (Cyclin D1), anti-apoptotic (Bcl-xl), and pro-apoptotic (Bax) proteins. Results demonstrated the existing chemo-resistance of CSCs (Fig. 6). ABCG2 was highly expressed in the untreated groups but decreased in the presence of sFRP4, doxorubicin/cisplatin, and combinatorial treatments, with the latter inducing the lowest expression levels. *c-Myc* had similar expression levels in all CSCs, except ovarian A2780-Cis CSCs (Fig. 6g). The levels of the proto-oncogene cyclin D1 decreased in all the combinatorial treatments of CSCs compared to untreated CSCs, except in prostate PC3 CSCs (Fig. 6c). Overexpression of the anti-apoptotic protein Bcl-xl in untreated CSCs confers chemo-resistance; however, the combinatorial treatment produced a significant decrease in protein expression levels, indicating sFRP4’s pro-apoptotic capacity. Expression of the pro-apoptotic protein Bax was lower in untreated CSCs but increased significantly with combinatorial treatment. The increased Bax/Bcl-xl expression level ratio confirms the pro-apoptotic role of sFRP4.

## Discussion

The role of CSCs in solid tumors is well established^20–22^. CSCs are in a quiescent state and remain in the resting phase of the cell cycle (G0 phase), expressing high levels of drug efflux transport systems^23, 24^. Due to the CSCs’ dormant state, chemotherapeutic drugs are unable to target CSCs, whereas they kill only proliferative tumor cells that are in M phase^25, 26^. Therefore, the knowledge of chemo-resistance associated with CSCs is of great importance for our understanding of tumors of the reproductive system (i.e. breast, prostate, and ovary), which are tumors with poor prognosis. In order to gain further insights into the chemo-sensitization of CSCs, we investigated the role of sFRP4 when used in combination with the chemotherapeutic drugs doxorubicin and cisplatin on CSCs derived from various tumor cell lines. Our study demonstrates sFRP4 alone or in combinatorial treatment with chemotherapeutic drugs elicits anti-proliferative effects, spheroid disruption, decrease in cell survival, and initiation of apoptosis within the CSCs, therefore indicating chemo-sensitization.

The CSCs were identified from breast (MDA231 and MCF7), prostate (PC3 and LnCap), and ovary (A2780 P, A2780 ADR, and A2780 CIS) tumor cell lines based on their ability to form spheroids in serum free conditions, elevated expression of CSC surface markers, high expression of ABC drug transporters (ABCG2), cell survival protein (Cyclin D1), oncogenes (c-Myc), and the ability to escape cell death/apoptosis (Bcl-xl). The resistance in CSCs is often correlated to the CSC surface marker profiles; CD44^+High^ and CD133^+High^ cells are highly radio-resistant in colon cancer and they have a higher DNA repair capacity and ability to escape apoptosis compared to CD44^Low^ and CD133^Low^ CSCs^27^. Targeting CD44^+^ cells demonstrated higher anti-proliferative activity *in-vitro* compared to the anti-tumor drug SN38 when used against colon, gastric, breast, esophageal, lung, and ovarian cancer cells^28, 29^. A reduction in the expression of CSC markers was observed in our study, where we demonstrated that sFRP4 in combination with chemotherapeutic drugs was able to decrease the CD44^+^ / 24^−^ population for breast-derived CSCs, and the CD44^+^ / CD133^+^ population for prostate and ovary-derived CSCs. In ovarian A2780 P derived CSCs, the cisplatin treatment aberrantly increased the CD133^+^ CD44^+^ population, and decreased the CD44^+^ and CD133^+^ alone population; this aberration could imply that phenotypic switching of CSCs has occurred, a process that is still not well understood^30^.

Clinically, CSCs reside in anatomically distinct regions within the tumor microenvironment (known as niches), which preserve the CSCs’ phenotypic plasticity, facilitate metastatic potential, and support high expression of drug efflux transporters, making them highly chemo-resistant^31^. Although CSC isolation and characterization has been studied extensively, *in-vitro* CSCs niches are characterized by spheroid forming capacity in serum free conditions^32^. We showed that targeting the Wnt signaling pathway by using sFRP4 has the capacity to disrupt the niches when sFRP4 was used in combination with chemotherapeutic drugs. Spheroid disruption by sFRP4 decreases the CSCs’ plasticity and cell-cell adhesion, initiating the CSCs’ differentiation towards tumor cells and reducing their self-renewal capacity. This opens the gateway for chemotherapeutic drugs to target the cells at high potency. We confirmed spheroid disruption using immunofluorescence, and we observed the spheroid disruption was associated with a reduced CSCs’ marker profile. We also observed a marked reduction of the proliferation marker Ki-67 and drug transporter ABCG2 in sFRP4 combinatorial treatment. The prostate cell line LnCap-derived CSCs showed an absence of CD44^+^ expression, which is in agreement with previous work^33^.

In previous studies, sFRP4 has shown an anti-proliferative capacity in CSCs derived from glioblastoma multiforme, and head and neck tumor^19, 34^. In this study we have demonstrated that sFRP4 in combination with chemotherapeutic drugs decreased the viability of CSCs compared to drug treatment alone, indicating sFRP4’s role in the increased chemo-response of CSCs.

The Wnt signaling pathway has been reported to regulate stemness in CSCs derived from colon cancer^35^ and breast cancer^36^, although Wnt activation is higher in breast CSCs compared to normal stem-like cells^37^. Genes controlling the stemness of CSCs have distinct functions and are important for CSC development and self-renewal, and are responsible for replicative quiescence^38^. We hypothesized that cancer stemness inhibition can effectively suppress metastatic potential and tumor recurrence, although gene profiling of cancer stemness is more similar to embryonic stem cells than adult stem cells^39^. In this study, we show that sFRP4 reduced the expression of various stemness genes including *Sox2, Klf4, Nanog*, and *Oct4* when treated in combination with chemotherapeutic drugs. These genes encode key stemness transcription factors that are important for maintenance of pluripotency^40, 41^. These data demonstrate the role of sFRP4 in inhibiting CSCs by modulating stemness gene expression.

Chemo-resistance of CSCs is due to alterations in expression of anti-apoptotic (Bcl-2 family) and pro-apoptotic (Bax) genes^42^. Apoptosis can be triggered by two pathways: (a) the extrinsic pathway where caspase-8 activation is initiated by the ligand of death receptors on the cell surface; and (b) the intrinsic pathway where mitochondria release *cytochrome c* ^43^. Release of *cytochrome c* is a crucial step that activates caspase-9 by assembling the apoptosome, further activating the downstream executioners caspase 3/7^44^. In previous studies, the relationship between sFRP4 and apoptosis has been identified, demonstrating sFRP4 as a pro-apoptotic agent^18, 45^. This was further confirmed by assessing the integrity of the mitochondrial membrane when the CSCs were treated with sFRP4. We observed the mitochondrial depolarization by application of the JC-1 assay, as low ∆ψ_M_ (mitochondrial membrane potential) due to depolarization is indicative of apoptosis, indicating sFRP4 chemo-sensitzation involves the initiation of apoptotic pathways. We further demonstrated the pro-apoptotic role of sFRP4 with an elevation in caspase 3/7 expression in CSCs treated with sFRP4 alone or in combination with chemotherapeutic drugs, indicating the later onset of apoptosis.

The overexpression of Bcl-2 is associated with chemo-resistance. The 3D protein structure of Bcl-xl revealed the structural similarities within the Bcl-2 family, possessing 4 BH domains and promoting cell survival by inactivation of Bcl-2 counterparts and preserving outer mitochondrial membrane integrity^46^. One of the early studies to show the chemo-sensitizing effects targeting Bcl-2 involved treating patients with Bcl-2 antisense (oblimersen sodium) in combination with chemotherapeutic drugs in chronic lymphocytic leukemia, leading to improved survival^47, 48^. We hypothesized that sFPR4 has the potential to bind the hydrophobic groove of anti-apoptotic Bcl-2, which would oligomerize Bax and can subsequently lead to mitochondrial membrane potential depolarization, releasing *cytochrome c*. We demonstrated a gradual increase in Bax expression and decreased Bcl-xl expression with sFRP4 and chemotherapeutic drug treatment alone; however, the combinatorial treatment elevated Bax and inversed the effect on Bcl-xl. The Bax/Bcl-xl ratio is an indicator for apoptosis, and an increased ratio depicts the activation of caspase 3^49^. The Bax/Bcl-xl ratio was consistently high in all CSCs treated with combinatorial treatment. The elevated expression of apoptotic genes within all the CSCs indicates sFRP4’s role as a pro-apoptotic agent.

We also observed a decrease in Cyclin D1 expression, which is an oncogene driving cell cycle progression. Cyclin D1 interacts with proteins involved with DNA repair, RNA metabolism, and cell structure; deregulation of Cyclin D1 will affect cellular processes and eventually lead to an inefficient DNA damage repair system^50^. In the Wnt signaling pathway, GSK-3β regulates Cyclin D1 degradation, and the gene expression is activated by Wnt signaling^51, 52^. SFRP4 is a Wnt antagonist, which binds to the frizzled receptors and activates the GSK-3β destruction complex, initiating the degradation of Cyclin D1. In contrast, *c-Myc* expression showed no decrease in A2780 CIS, PC3, and MCF7-derived CSCs. c-Myc is an oncoprotein that is an important regulator in stem cell biology^53^ and correlates to tumor metastasis^39^. CSCs exhibit a high expression of c-Myc, and downregulation of c-Myc leads to apoptosis under various circumstances^54–57^. We hypothesize that a different dose of sFRP4 and chemotherapeutic drugs would enable a reduction in *c-Myc* expression.

In summary, sFRP4 chemo-sensitizes CSCs derived from breast, prostate, and ovary tumor cell lines by reducing their pro-oncogenic profile, stemness capacity, cell survival protein and oncogene expression, making them more responsive to chemotherapeutic drugs. Chemo-sensitization by sFRP4 *in vivo* may decrease the required chemotherapeutic load required to reduce the tumor mass. SFRP4 prevents a sustained Wnt inhibition in order to provide a therapeutic window for chemotherapy while sparing normal Wnt-dependent tissues. Further *in vivo* studies may confirm the role of sFRP4 in the chemo-sensitization of CSCs to prevent tumor relapse and lead to tumor resolution.

## Materials and Methods

### Monolayer cell culture

Cell culture plates for adherent cells were purchased from Nunc™ (ThermoFisher Scientific). The human breast cells line MDA-MB 231 (ER-) and MCF-7 (ER+), human ovary cell lines A2780-P, A2780-ADR, and A2780-Cis, and human prostate cell lines PC-3 (AR−/PSA−) and LnCap (AR+) were purchased from American Type Culture Collection (ATCC, USA). The cells were cultured in RPMI-1640 medium (Gibco #11875–093) supplemented with 10% fetal bovine serum (Bovogen #SFBS) and 100 U/ml PenStrep (Life Technologies #15070063). All cells were maintained at 37 °C in a humid incubator with 5% CO_2_.

### Cancer stem cell isolation

For CSC isolation, culture plates with an ultra-low-attachment surface were purchased from Corning Life Sciences. CSCs were cultured in serum-free medium (SFM) containing basal medium RPMI-1640 + DMEM-HG (HyClone, USA #SH30081.02) supplemented with the growth factors bFGF (20 ng/ml) (ProSpec Bio #cyt-085), EGF (20 ng/ml) (ProSpec Bio #cyt-217), and 1× B27 (Gibco #17504044), and 100 U/ml PenStrep (Life Technologies #15070063). CSC-enriched populations of cells were obtained by plating a single cell suspension of breast, ovary, and prostate tumor cells at 10000 cells/cm² in SFM on Low-adherent six-well plates (Corning #3471). CSCs were isolated in SFM; the spheroids are formed at 3^rd^ Day of plating tumour cells. SFRP4 (250 pg) and chemotherapeutic drugs alone or in combination were added on the 3^rd^ Day for 24 hours. Post 24 hour treatment; the spheroids were dissociated and maintained in CSC culture medium in low-attachment-surface plates for functional studies.

### Chemo-Sensitization/Drug treatment

The drugs used in this study were purified sFRP4 (R&D Systems #1827-SF-025), doxorubicin (Sigma #D1515), and cisplatin (Sigma #P4394). CSC sensitization with sFRP4 was performed by adding sFRP4 to the cell culture at 250 pg/ml^58^ for 24 hr at 37 °C in 5% CO_2_ incubator. Doxorubicin was tested at an IC50 value of 1–10 μM. Cisplatin was tested at its IC50 value of 10–50 μM. The drug treatment for the downstream analysis was optimized at 250 pg of sFRP4 alone or in combination with 5 μM of doxorubicin (breast tumor cells only) and 30 μM of cisplatin (ovary and prostate tumor cells only) for 24 hr. An MTT assay was used for the analysis of cellular viability.

### Cell Surface Markers

To assist in determining their identity, cell surface markers (Table 2) were examined in both monolayers and CSCs by flow cytometry (BD FACSCANTO II) using CellQuest data acquisition and analysis software. APC-CD44 (1:100) (BioLegend #338805), PE Cy7-CD24 (1:10) (BioLegend #311119), and PE-CD133 (1:100) (BioLegend #372803). Cells incubated with conjugated irrelevant IgGs were used as negative controls.

**Table 2:** Tumor specific cancer stem cell markers.

Cancer	CSC Markers	Reference
Breast	CD24^−/low^/CD44^+^	3, 59
Ovary	CD133^+^/ALDH1^+^	60
Prostate	CD133^+^/CD44^+^/ABCG2^+^	61

### Immunofluorescence staining

The spheroids were plated on Poly-D-lysine pre-coated 96 well plates (Sigma #6407), incubated at 37 °C for 3 hr, and then fixed in 4% paraformaldehyde (Sigma #P6148) overnight at 4 °C. The cells were washed three times with PBS, incubated for 1 hr in 1% BSA Blocking buffer, and incubated with primary antibodies CD44 (Cell Signaling #3570); CD24 (ThermoFisher #MA5-11828); CD133/1 (Miltenyi #130-090-422); ABCG2 (Cell Signaling #42078); Ki-67 (Millipore #AB9260) overnight at 4 °C. After three 10 mins washes with PBS, the cells were incubated with the appropriate secondary antibody (see Supplementary Table 1) for 1 hr at room temperature. After three 10 min PBS washes, cells were incubated with Hoechst 33342 (Sigma #14533) at 1:20000 dilution at room temperature for 15 min. The cells were then washed with PBS three times for 5 min each and observed using an Ultraview Vox spinning disk confocal microscope (Perkin Elmer).

### MTT Viability Assay

#### Cell viability kit

An MTT kit (Sigma #M5655) was used to measure cell metabolic viability.

#### Monolayers

5000 cells/cm² were plated in a flat-bottomed 96-well plate for 2 days with culture medium. After that, sFRP4 alone or in combination with the tumor-specific drug was added and the cells were incubated for 24 hr. After drug treatment, the MTT assay was performed as per the manufacturer’s instructions.

#### CSCs

10000 cells/cm² of monolayer cells were plated in a low-adherent flat-bottomed 96-well plate (Corning #3474) for 3 days in non-adherent SFM conditions. After that, sFRP4 alone or in combination with the tumor-specific drug treatment was added and the cells left for 24 hr, following which the MTT assay was performed. Plates were read at 595 nm using an EnSpire Multilabel Plate Reader (Perkin-Elmer).

### Reverse transcription-polymerase chain reaction

Total RNA was isolated from cells using TRIzol reagent (Life Technologies #15596026) followed by chloroform extraction, isopropanol precipitation, and a 75% (v/v) ethanol wash. RNA samples (1 μg) were reverse-transcribed to cDNA using a High Capacity cDNA kit (Applied Biosystems #4368814). cDNA in 1 μl of the reaction mixture was amplified with PCR Master Mix (Life Technologies #K0171) and 10 μmol each of the sense and antisense primers. The thermal cycle profile was as follows: denaturation at 95 °C for 30 s, annealing at 55–61 °C for 30 s depending on the primers used, and extension at 72 °C for 90 s. Each PCR reaction was carried out for 35 cycles, and the PCR products were size fractionated on 1% agarose gel/GelGreen (1:10000) (Fisher Biotec #41005) and visualized under UV Trans illumination (FB Biotech). The primer sequences are described in Supplementary Table 2.

### Western Blotting

Cells were washed twice with PBS and then lysed in RIPA lysis buffer (Sigma #R0278) (150 mM NaCl, 1.0% IGEPAL^®^ CA-630, 0.5% sodium deoxycholate, 0.1% SDS, 50 mM Tris, pH 8.0, Proteinase Inhibitor 1x). Post sonication, cell lysates were centrifuged at 14000 g for 10 min at 4 °C, and the supernatants were used for Western blotting. The lysates were resolved by sodium dodecyl sulfate-polyacrylamide gel electrophoresis, transferred onto nitrocellulose membranes, and then stained with 0.1% Ponceau S solution (Sigma #P3504) to ensure equal loading of the samples. After being blocked with 5% non-fat milk for 60 min, the membranes were incubated with primary antibodies ABCG2 (Cell Signaling #42078); c-Myc (Cell Signaling #5605); Cyclin D1 (Abcam #ab134175); Bcl-xL (Cell Signaling #2764); Bax (Cell Signaling #5023); β-Actin (Cell Signaling #4970) overnight, and the bound antibodies were visualized with horseradish peroxidase-conjugated secondary antibodies using the ECL Western Blotting Substrate (Amersham, GE #RPN2106) on a Chemi-Doc (Bio-Rad) imaging analyzer. The Primary antibodies concentrations are described in Supplementary Table 3.

### Apoptosis Assays

#### JC-1 Assay

∆ψ_M_ is an important parameter of mitochondrial membrane and has been used as an indicator of cell health. JC-1 enters the mitochondria and changes its fluorescent properties based on aggregation of the probe, and forms complexes known as J-aggregates with intense red fluorescence. High ∆ψ_M_ predicts healthy cells and low ∆ψ_M_ exhibits mitochondrial membrane potential depolarization indicative of apoptosis. JC-1 activity was measured using JC-1 Mitochondrial Membrane Potential Assay Kit (Cayman #10009172). CSCs were enriched in 96 well low attachment plates, and treated with sFRP4 alone and in combination with chemotherapeutic drugs for 24 hr and compared with untreated CSCs. Post-treatment, 10 μl of JC-1 staining solution was added to each well and incubated at 37 °C for 30 min. The plate was washed with assay buffer at 400 g for 5 min twice. This was followed by addition of 100 μl of assay buffer to each well and read using an EnSpire Multilabel Plate Reader (Perkin-Elmer) for analysis. JC-1 aggregates were measured at excitation and emission wavelengths of 535 nm and 595 nm respectively. JC-1 monomers were measured at excitation and emission wavelengths of 485 nm and 535 nm. The ratio of fluorescent intensity of J-aggregates and J-monomers (Red: Green) was used as an indicator of cell health. The JC-1 assay kit is highly light sensitive and all procedures were conducted in dark conditions.

#### Caspase-3 Assay

Caspase-3 activity was measured using the EnzChek Caspase-3 Assay Kit II (Molecular Probes #E13184). Briefly, 50 μl of the supernatant was added to an individual well of a 96-well micro fluorescent plate and incubated for 10 min at room temperature. After incubation, 50 μl of the 2× working substrate (5 M Z-DEVD-R110) were added to each well and further incubated for 30 min at 37 °C. Fluorescence was measured at 485 nm excitation and 538 nm emissions using an EnSpire Multilabel Plate Reader (Perkin-Elmer). Caspase-3 activity was expressed as arbitrary units of fluorescence.

### Statistics

Statistical analysis was performed with GraphPad Prism V5.0 (GraphPad software, La Jolla, USA) using one-way ANOVA for analysis variance with Bonferroni test for comparison showing significance as ***P < 0.001; **P < 0.01; *P < 0.05. Data are presented as mean ± standard error of mean.

### Data Availability

The datasets generated during and/or analysed during the current study are available from the corresponding author on reasonable request.

## Electronic supplementary material

Supplementary Dataset 1
