# Supplementary material for: Secreted Frizzled-related protein 4 (sFRP4) chemo-sensitizes cancer stem cells derived from human breast, prostate, and ovary tumor cell lines
Source: Sci Rep. 2017 May 23;7:2256. doi: 10.1038/s41598-017-02256-4 (PMC5442130; doi:10.1038/s41598-017-02256-4)
Supplement: Supplementary file 1 — Supplementary Dataset 1 [file 41598_2017_2256_MOESM1_ESM.doc]

# **Title:** Secreted Frizzled-related protein 4 (sFRP4) chemo-sensitizes cancer stem cells derived from human breast, prostate, and ovary tumor cell lines

**Authors:** A. Deshmukh, S. Kumar, F. Arfuso, P.Newsholme, A. Dharmarajan*

**a**


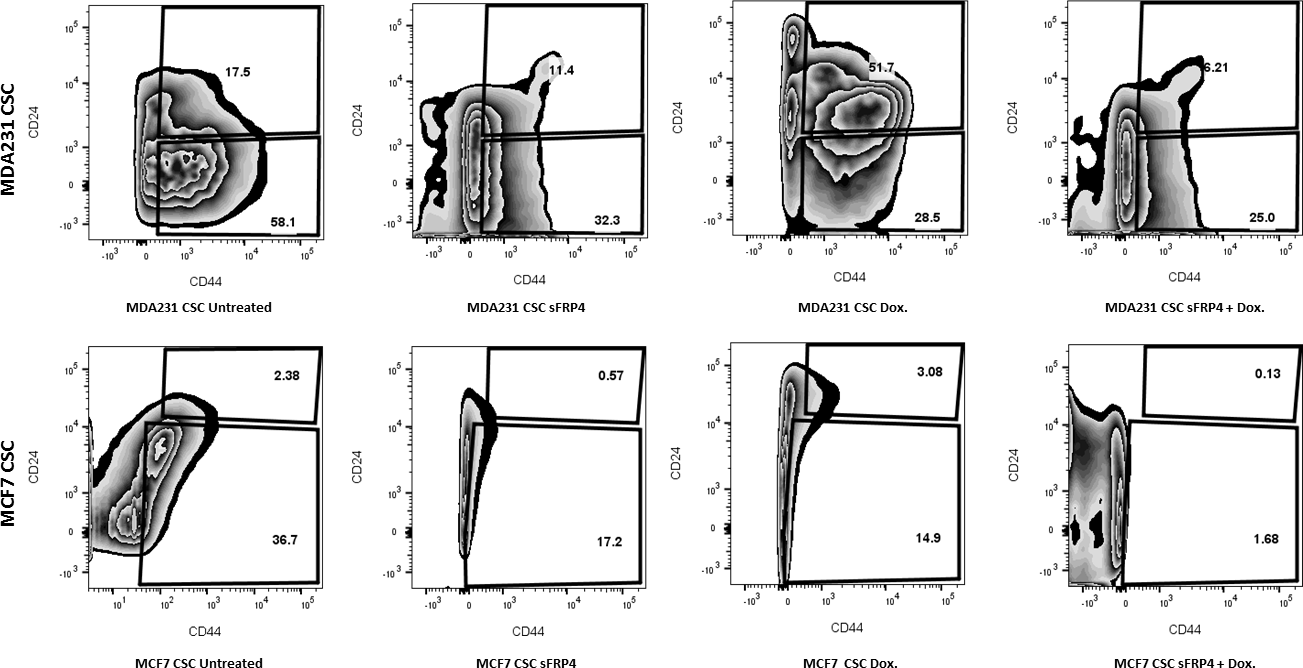

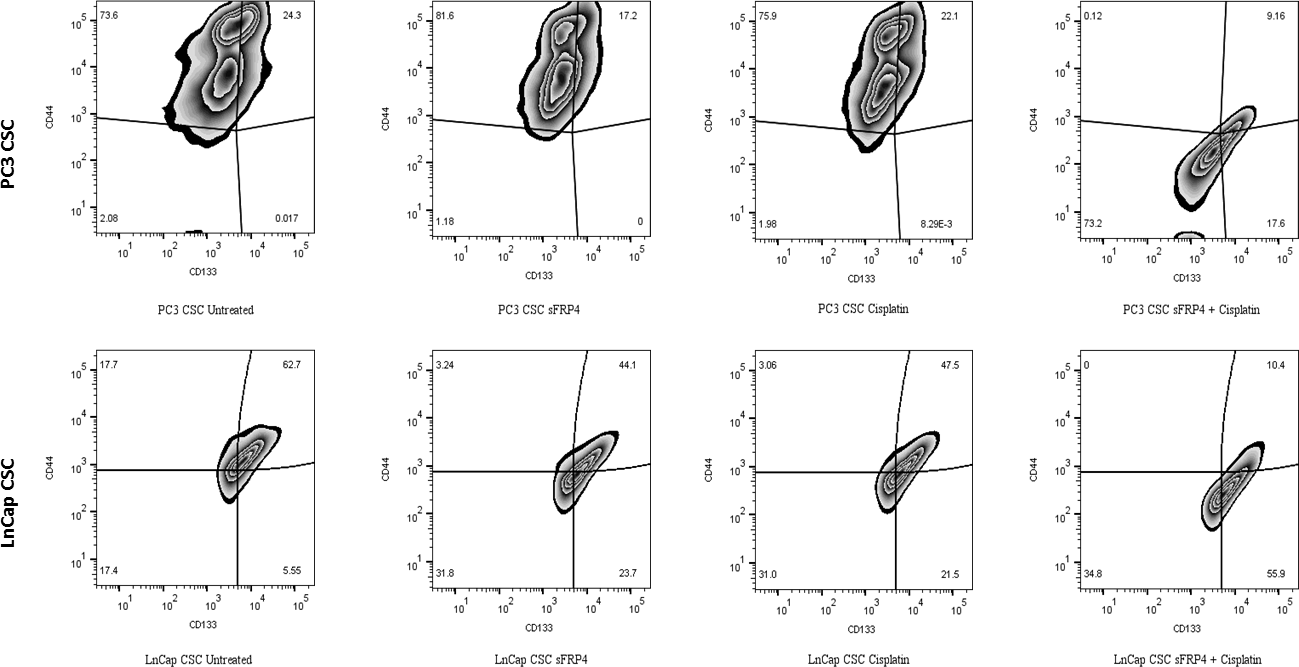


**b**

**c**


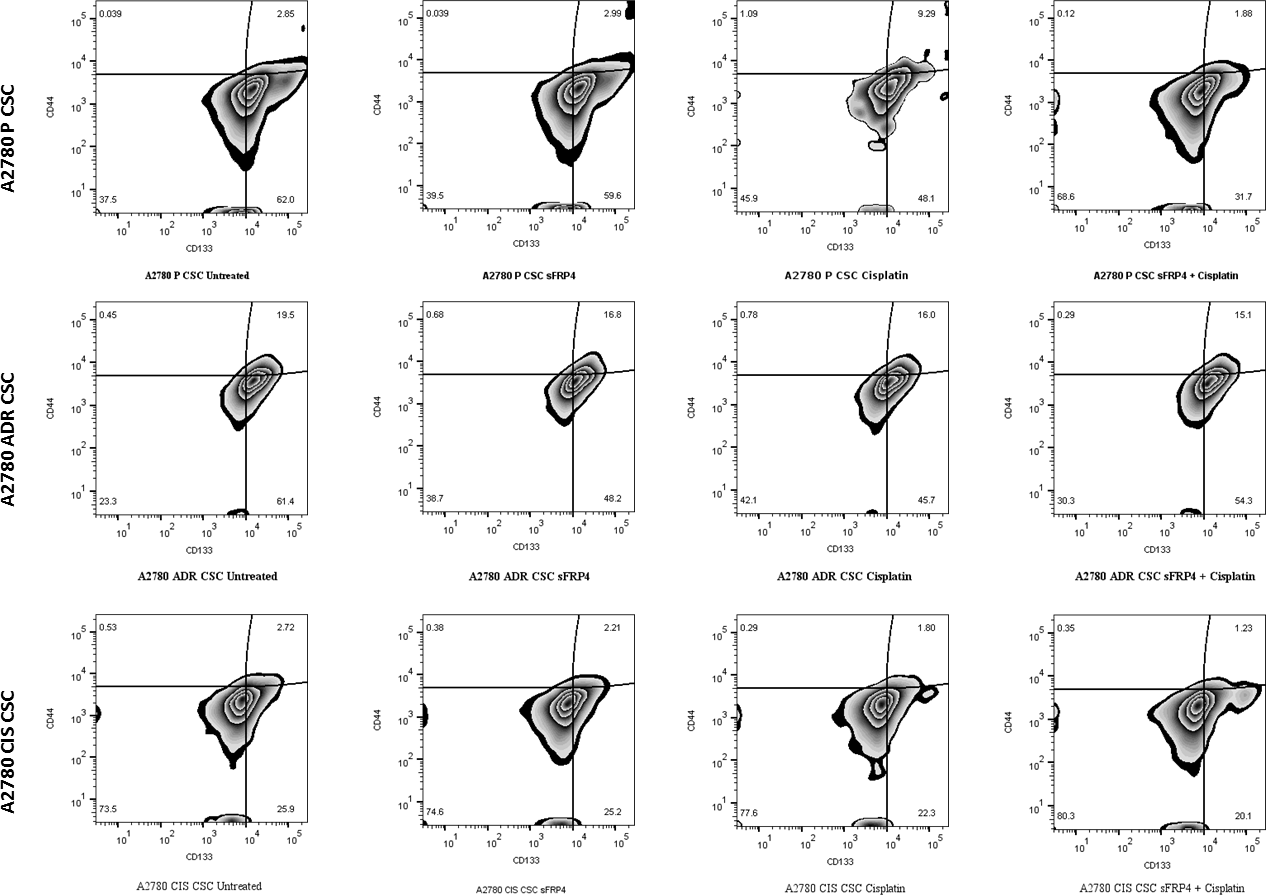


**CD133⁺/ CD⁺ (%)**

**Supplementary Figure 1**: Effect of sFRP4 on CSC characterisation: Using flow cytometry a) CD44/CD24 were used to detect breast-derived CSCs, b/c) CD133/ CD44 for ovary and prostate-derived CSCs. sFRP4 in combination with doxorubicin/ cisplatin reduced the CD44/24/133 CSC population.

**Supplementary Table 1:** Primary antibodies used for Immunofluorescence

33342

| **Primary Antibody (Clone)** | **1°**  **Dilution** | **Catalogue No.** | **Secondary Antibody** | **2° Dilution** |
| --- | --- | --- | --- | --- |
| **CD44** Cell Signaling Anti-mouse IgG, HRP-  1:300 1:10000  (156-3C11) #3570 linked | | | | |
| **CD24** ThermoFisher Anti-mouse IgG, HRP-  1:300 1:10000  (SN3) #MA5-11828 linked | | | | |
| **CD133/1** Miltenyi Anti-rabbit IgG, HRP-  1:100 1:10000  (AC133) #130-090-422 linked | | | | |
| **ABCG2** Cell Signaling Anti-rabbit IgG, HRP-  1:300 1:10000  (D5V2K) #42078 linked | | | | |
| **Ki-67** Millipore Anti-rabbit IgG, HRP-  1:500 1:10000  **(na)** #AB9260 linked | | | | |
| **Hoechst** Sigma | | | | |

1: 20000

#14533

-na- -na-

**Supplementary Table 2**: Primer sequences for CSC stemness markers

R- 5’ GTTGCTGTTGAAGTCACAGGAGAC 3’

| **Genes** | **Primers** | **Base Pair (bp)** | **Annealing Temperature**  **°C** |
| --- | --- | --- | --- |
| F- 5’ CCATCCACACTCACGCAAAA 3’  Sox2 139 59  R- 5’ TATACAAGGTCCATTCCCCCG 3’ | | | |
| F- 5’ TCCCATGCATTCAAACTGAGG 3’  Oct4 103 60  R- 5’ CCAAAAACCCTGGCACAAACT 3’ | | | |
| F- 5’ TGGACACTGGCTGAATCCTTC 3’  Nanog 142 59  R- 5’ CGTTGATTAGGCTCCAACCAT 3’ | | | |
| F- 5’ CTGCGGCAAAACCTACACAA 3’  KLF4 182 60  R- 5’ GGTCGCATTTTTGGCACTG 3’ | | | |
| F- 5’ CAGAACATCATCCCTGCATCCACT 3’  GAPDH 185 61 | | | |

**Supplementary Table 3**: Primary and secondary antibodies used in Western blotting to measure relative protein expression

(13E5)

| **Primary Antibody (Clone)** | **1°**  **Dilution** | **Molecular Weight (Kda)** | **Catalogue No.** | **Secondary Antibody** | **2°**  **Dilution** |
| --- | --- | --- | --- | --- | --- |
| **ABCG2** Cell Signaling Anti-rabbit IgG,  1:1000 65-80 1:2000  (D5V2K) #42078 HRP-linked | | | | | |
| **c-Myc** Cell Signaling Anti-rabbit IgG,  1:1000 57-65 1:2000  (D84C12) #5605 HRP-linked | | | | | |
| **Cyclin D1** Abcam Anti-rabbit IgG,  1:10000 34 1:2000  (EPR2241) #ab134175 HRP-linked | | | | | |
| **Bcl-xL** Cell Signaling Anti-rabbit IgG,  1:1000 30 1:2000  (54H6) #2764 HRP-linked | | | | | |
| **Bax** Cell Signaling Anti-rabbit IgG,  1:1000 20 1:2000  (D2E11) #5023 HRP-linked | | | | | |
| **β-Actin** Cell Signaling Anti-rabbit IgG, | | | | | |

1:1000 45

#4970

HRP-linked

1:2000
